# Dizziness in Parkinson’s disease patients is associated with vestibular function

**DOI:** 10.1038/s41598-021-98540-5

**Published:** 2021-09-23

**Authors:** Jeong-Ho Park, Suk Yun Kang

**Affiliations:** 1 Department of Neurology, College of Medicine, Soonchunhyang University Bucheon Hospital, Bucheon, Republic of Korea; 2 Department of Neurology, Dongtan Sacred Heart Hospital, Hallym University College of Medicine, 7, Keunjaebong-gil, Hwaseong, Gyeonggi-do 18450 Republic of Korea

**Keywords:** Diseases, Medical research, Neurology, Signs and symptoms

## Abstract

Dizziness is common in Parkinson’s disease (PD) patients. It is known that orthostatic hypotension (OH) is the main cause of such dizziness, but even without OH, quite a few PD patients complain of dizziness in the clinic. It can be regarded as non-specific because most of these patients have no neurological abnormalities. We hypothesized that this type of dizziness would be associated with vestibular function, although included patients did not have clinically confirmed vestibulopathy. We studied 84 patients without OH among 121 PD patients. Their clinical features and function were compared between patients with and without dizziness. Hoehn and Yahr stage (H&Y stage), the Unified Parkinson's Disease Rating Scale (UPDRS) part III, the Korean version of the Mini-Mental State Examination (K-MMSE), education years, disease duration, total levodopa equivalent daily dose (LEDD), the presence of dizziness, the dizziness severity, and orthostatic hypotension were tested. Vestibular evoked myogenic potentials (VEMPs) were used to characterize vestibular function. Ocular (oVEMPs) and cervical (cVEMPs) were recorded. oVEMPs in the right side showed significantly reduced potentials (p = 0.016) in PD patients with dizziness, but cVEMPs did not (all ps > 0.2). Bilateral absent oVEMP responses were more common in PD patients with dizziness (p = 0.022), but the frequencies of bilateral absent cVEMP responses were not different between the dizzy and non-dizzy groups (p = 0.898). Dizziness in PD patients without orthostatic hypotension may be associated with vestibular hypofunction. Our results provide evidence that can aid clinicians when making a treatment plan for patients with dizziness. i.e., strategies to enhance reduced vestibular function may be helpful, but this suggestion remains to be evaluated.

## Introduction

Dizziness is a common complaint in Parkinson’s disease (PD) patients, and its prevalence ranges 48–68%^1^. This dizziness is mainly due to orthostatic hypotension (OH), but a high proportion of dizzy PD patients without OH are seen in the clinical setting. Even they do not show postural instability and ataxia. One study reported that non-specific dizziness was seen in 29.7% of patients in the early stage of PD. It is the second most common type of dizziness after orthostatic hypotension^1^. Dizziness has also been reported to be a prodromal symptom of PD. PD patients report frequent dizziness at least 5 years before diagnosis^2^.

Vestibular dysfunction is seen in PD patients^3^. Neuropathological changes in the vestibular nucleus complex, prepositus hypoglossi, and the root of the vestibular nerve have been reported^3,4^. The vestibular evoked myogenic potential test (VEMP) is a detailed electrophysiological test that evaluates the vestibular system^5^. However, the previous results of VEMPs in PD patients have been inconsistent^6–9^, maybe due to different study population characteristics. We hypothesized that PD patients with dizziness might have more severe vestibular dysfunction, and VEMPs might reflect their dizziness symptoms. Therefore, we aimed to investigate if VEMPs differed between PD patients with and without dizziness who did not present with orthostatic hypotension.

## Results

Eighty-four of 121 patients were analyzed. Thirty-seven patients were excluded because orthostatic hypotension was documented in positional blood pressure measurement or the head-up-tilt table test (17 patients) or was not evaluated (20 patients). Vestibular function in the neurological examination was normal in all participants.

Demographics and clinical features between patients with and without dizziness are summarized in Table 1. Except for the sex ratio, these variables were not different between the groups.

**Table 1:** Clinical characteristics of patients without dizziness and with dizziness.

	Without dizziness n = 56	With dizziness n = 28	p* value
Age, years	64.4 ± 8.7	67.6 ± 9.2	0.126
Women, n (%)	37 (66.1)	10 (35.7)	0.011
Duration of disease, years	3.3 ± 3.1	3.5 ± 2.4	0.760
K-MMSE	26.2 ± 3.2^a^	25.8 ± 3.0	0.637
Education, years	8.8 ± 5.3^b^	8.9 ± 4.7^c^	0.946
HY stage	1.8 ± 0.7	2.1 ± 0.7	0.095
UPDRS III	16.1 ± 8.8	17.5 ± 7.6	0.471
Total LEDD	389.0 ± 303.0	381.3 ± 340.2	0.916
Dizziness severity	-	3.8 ± 2.1^d^	-

K-MMSE, the Korean version of the Mini-Mental State Examination; HY stage, Hoehn and Yahr stage; UPDRS III, Unified Parkinson’s Disease Rating Scale part III; Total LEDD, total levodopa equivalent daily dose.

Values are expressed as means ± SD or number (percentage). Dizziness severity was evaluated with visual analogue scale having a range of scores from 0 to 10. Missing cases were not included and the number of the case was ^a^two, ^b^four, ^c^one, and ^d^three.

*p < 0.05 indicates significant differences.

**Figure 1:**
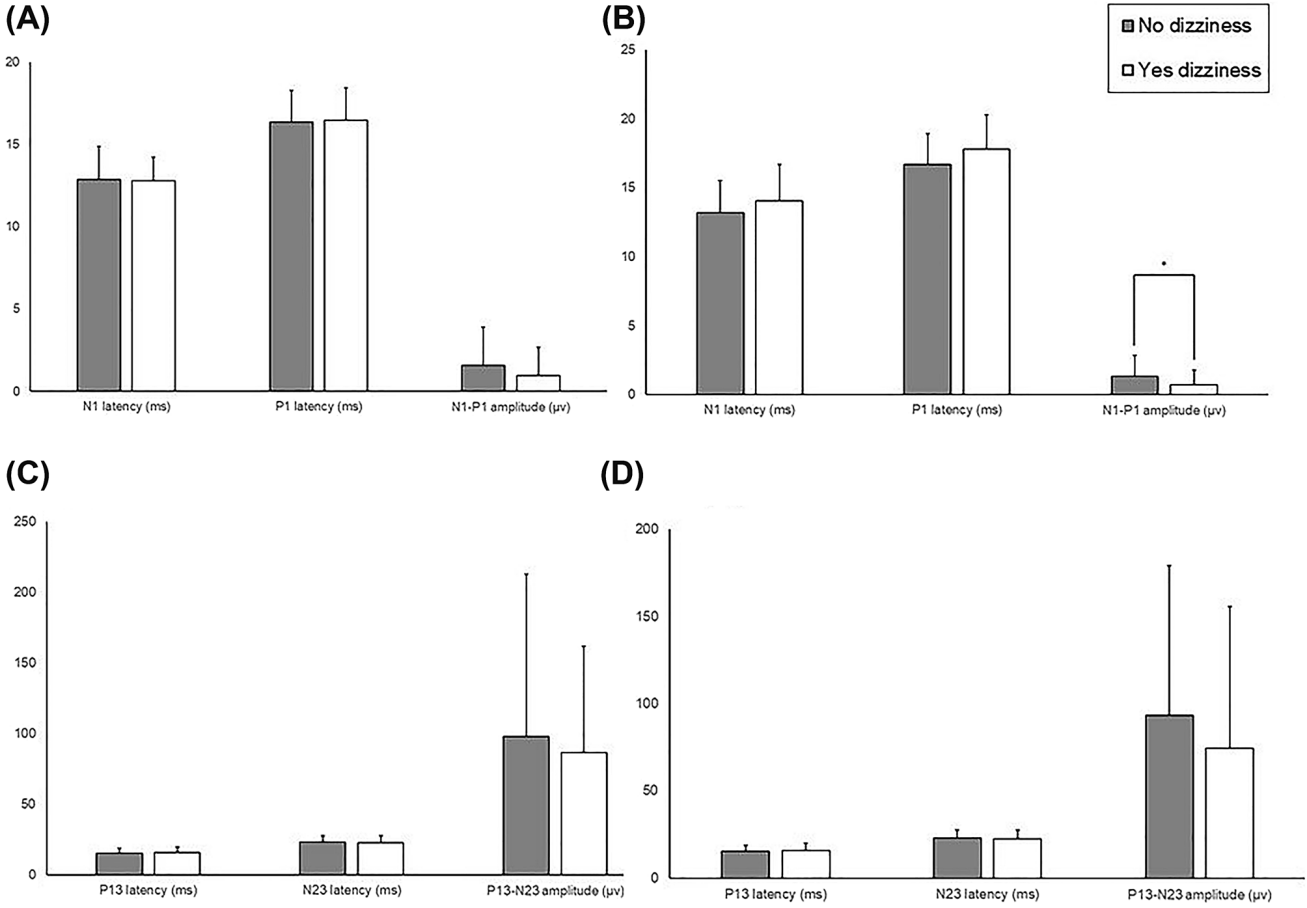
The latencies and peak-to-peak amplitudes of VEMPs in PD patients with and without dizziness. Ocular VEMPs in the left (**A**) and the right side (**B**). Cervical VEMPs in the left (**C**) and the right side (**D**).

For the left ocular VEMP (oVEMP), the mean values of the N1-P1 amplitude, and the N1 and P1 latencies were not statistical different by dizziness group (p = 0.327, 0.515, 0.835, respectively, Fig. 1A). For the right oVEMP, the N1-P1 amplitude was significantly lower in dizzy patients (p = 0.016, Fig. 1B). The N1 latency and P1 latency were longer, but not statistically (p = 0.308, 0.244, respectively). There were no differences in cervical VEMP (cVEMP) parameters between the two groups (all p’s > 0.2, Fig. 1C,D).

**Figure 2:**
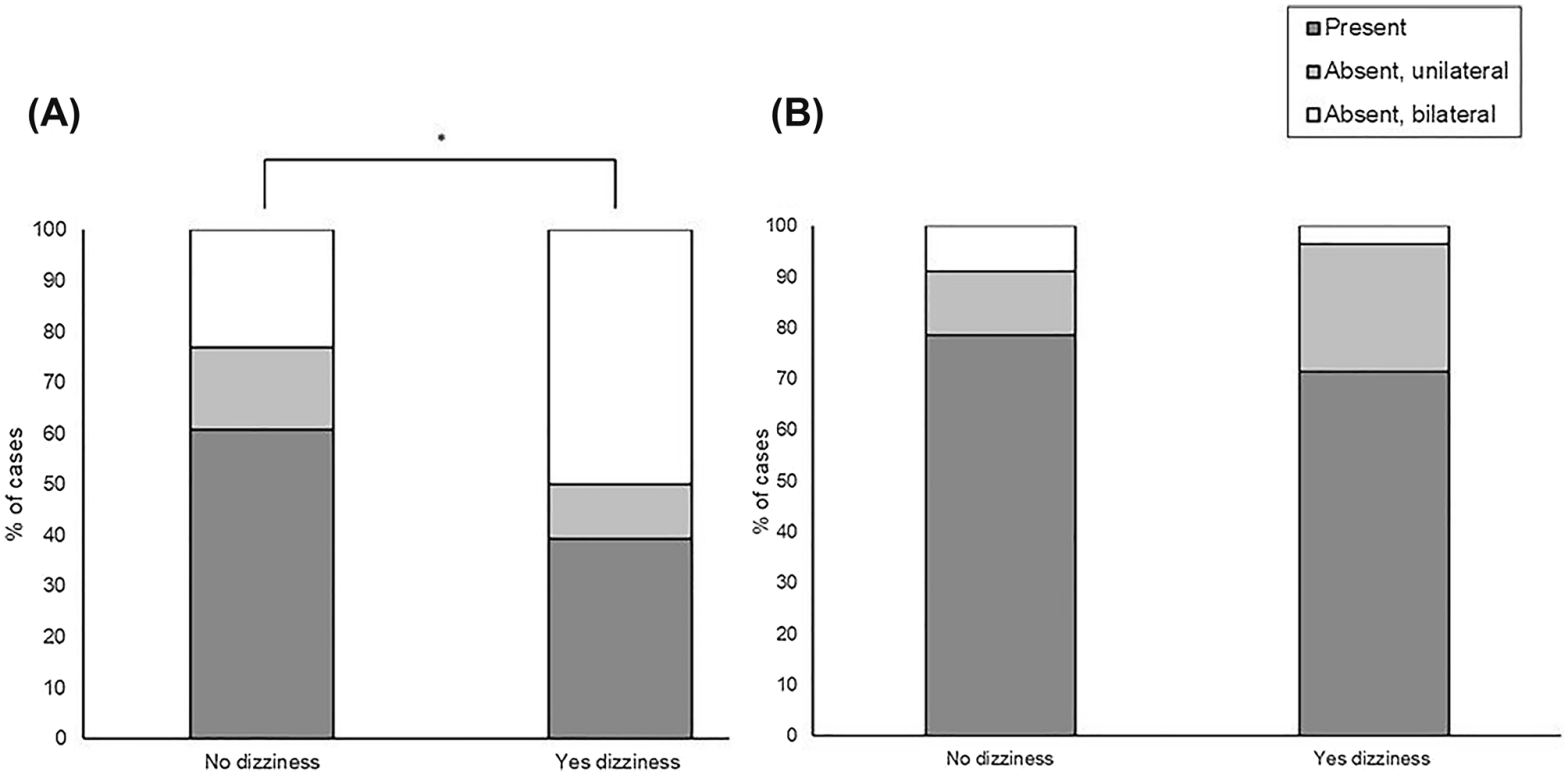
The comparison of bilateral absent, unilateral absent, and present VEMP responses between PD patients with and without dizziness. (**A**) oVEMPs. (**B**) cVEMPs.

The ratio of the bilateral absent group was higher in dizzy patients for oVEMPs (p = 0.022), but no difference was seen in cVEMPs (p = 0.898, Fig. 2).

The scores for dizziness severity were obtained in 25 patients with dizziness. There were no significant correlations of dizziness score with VEMP parameters (Table 2). The N1-P1 amplitudes of the left oVEMPs were negatively correlated with dizziness severity but not statistically significantly (r = − 0.379, p = 0.082).

**Table 2:** Partial correlations between the dizziness severity and VEMP parameters.

Parameter	Correlation coefficient	p*
**oVEMP**		
Left		
N1 latency	− 0.052	0.903
P1 latency	− 0.282	0.498
N1-P1 amplitude	− 0.379	0.082
Right		
N1 latency	0.569	0.141
P1 latency	0.330	0.425
N1-P1 amplitude	− 0.131	0.560
**cVEMP**		
Left		
P13 latency	0.018	0.937
N23 latency	− 0.080	0.729
P13-N23 amplitude	− 0.205	0.360
Right		
P13 latency	− 0.068	0.803
N23 latency	− 0.245	0.361
P13-N23 amplitude	− 0.199	0.374

*p < 0.05 indicates a significant correlation.

## Discussion

Our study suggests that PD patients with dizziness without orthostatic hypotension may have lower vestibular functions than PD patients without dizziness. Our results showed that the amplitudes of oVEMPs were smaller, and bilaterally absent oVEMP responses were more common in PD patients with dizziness. There was no statistical difference in cVEMP responses between patients with and without dizziness.

Several studies previously reported abnormal VEMP responses in PD patients^6–11^, suggesting brainstem involvement^8,11^, and some associated motor and non-motor symptoms (NMSs) with the VEMP findings, but these associations seemed to be inconsistent^6,7,9–11^. The vestibular dysfunction was related to postural instability^10,12^, but the others were not^9,13^. Other NMSs disorders were also associated with VEMP responses, including sleep, perception, cognition, urinary problems, cardiovascular, and sexual dysfunction, although seemingly unrelated to vestibular function^6,9,10^. It was explained by the degeneration of several brainstem nuclei responsible for the motor and NMSs^9^, and by the VEMP circuit that spans the midbrain, the pons, and the medulla^14,15^. Considering our results that patient dizziness may result from vestibular dysfunction and the fact that VEMPs are useful to evaluate the vestibular system, we think that it may be clinically reasonable that VEMPs reflect dizziness rather than other NMSs in PD patients. Disappointingly, our study did not find correlations between dizziness level and VEMP parameters. This may be due to the use of too simple methods to measure severity accurately. On the contrary, the VEMPs were reported to be correlated with the other NMSs^6,9,10^.

oVEMP responses were different between patients with and without dizziness, but cVEMP responses were not different between these groups in our study. The reason for the different responses is unclear. VEMPs are short-latency, otolith-dependent reflexes recorded from the eye (oVEMPs) and neck muscles (cVEMPs)^15^. The anatomical pathways of these two VEMPs are thought to be different. The oVEMP pathway presumably starts from the vestibular nucleus, crosses the midline, and ascends through the medial longitudinal fasciculus (MLF) at the upper pons and midbrain level, then to the ocular motor nuclei. The cVEMP presumably involves a descending pathway from the vestibular nucleus, ipsilateral medial vestibulospinal tract in the pons and medulla, then to the accessory motor neurons^14,15^. It has been suggested that upper brainstem function might be involved and lower brainstem function preserved in early PD patients^8^. However, the changes in oVEMP and cVEMP responses were not different in another study^10^. Besides, because it is thought that the earliest PD-related pathology appears in the olfactory bulb and dorsal vagal nucleus, lower brainstem regions also appear to be involved in early PD patients. Therefore, further studies are needed to determine whether there is a difference in clinical significance between oVEMP abnormality alone or both oVEMPs and cVEMPs.

This study has some limitations. First, we did not investigate other NMSs such as rapid eye movement sleep behavior disorder that could affect the VEMP responses^9,10^. Second, because our study also included patients with more than H&Y stage 2, and it could be supposed that dizziness would be more common in patients with greater H&Y stage, it can be argued that this could affect our results. However, there was no statistical difference in the number of patients with and without dizziness among patients with an H&Y score of 2.5 or higher. (P = 0.137, Supplementary Table 2). Third, there was no sophisticated instrumental assessment of vestibular function (caloric tests, electro- or video nystagmography, dynamic visual acuity, posturography, etc.). Detailed neurological examination may not be enough to examine the functioning of the human vestibular system. Forth, it can be argued that the definition of dizziness is vague. However, as I described in the introduction, we can see that patients with dizziness do not have any abnormality on neurologic examination or instrumental assessment in clinical practice. Until now, we do not seem to have a good understanding of the cause or mechanism of such dizziness. This was also the reason why this study was started. Fifth, we used a 10-point visual analogue scale to evaluate the severity of dizziness. If we would have evaluated the severity in detail, which could be very informative. However, patients with PD had to be evaluated as soon as possible to avoid exhaustion because they had motor problems. Sixth, since the only difference between patients with and without dizziness is the N1-P1 amplitude for the right oVEMP, we should be more cautious in interpreting results. However, because the ratio of the bilateral absent oVEMP group was higher in patients with dizziness, I think the results of oVEMP may have meaning.

In conclusion, among patients with dizziness, patients whose dizziness was not caused by orthostatic hypotension, and perhaps most of those diagnosed with non-specific dizziness, appear to have a poor vestibular function. Our results suggest that treatment to enhance vestibular function, such as vestibular stimulation, may be helpful in such patients, which requires further study^3,12,13^. Our results also show that studies on clinical progress and prognosis in dizziness patients with vestibular hypofunction are needed.

## Methods

### Patients

We prospectively screened 121 patients with PD who visited our hospital from 2015 to 2018 for this study. PD was diagnosed according to UK Brain Bank criteria^16^. Hoehn and Yahr stage (H&Y stage), the Unified Parkinson's Disease Rating Scale (UPDRS) part III, the Korean version of the Mini-Mental State Examination (K-MMSE), education, disease duration, total levodopa equivalent daily dose (LEDD), the presence of dizziness, and orthostatic hypotension tests were administered. Orthostatic hypotension was defined as a sustained reduction in systolic blood pressure of at least 20 mmHg or diastolic blood pressure of 10 mmHg within 3 min of standing or head-up tilt to at least 60° on a tilt table^17^. Patients were excluded if they had orthostatic hypotension or peripheral vestibulopathy on neurological examination. A detailed neurological examination was performed to exclude any other neurological disorders. The detailed neurological examination was summarized in supplementary Table 1.

This study limited dizziness to chronic dizziness without vestibular dysfunction and postural imbalance on neurological examination. It is similar to chronic subjective dizziness in the literature^18^ and has recently been incorporated into persistent postural-perceptual dizziness (PPPD). It is a syndrome, and its main features are non-spinning vertigo and perceived unsteadiness^19,20^. We slightly modified the diagnostic criteria of PPPD for the current study; (1) one or more symptoms of dizziness, unsteadiness, or nonspinning vertigo are present on most days for more than 3 months, (2) persistent symptoms occur without specific provocation but are exacerbated by upright posture, active or passive motion changes, and exposure to moving visual stimuli or complex visual patterns, (3) precipitation by conditions that cause vertigo, unsteadiness, dizziness, or imbalance, (4) normal neurological examinations for vestibular function and ataxia (no other neurological problems except parkinsonism). The severity of dizziness was rated using a 10-point visual analogue scale (0: no dizziness; 10: worst dizziness). All experiment protocols were approved by our Institutional Review Board of Soonchunhyang University Bucheon Hospital. All methods were carried out in accordance with relevant guidelines and regulations. Informed consent was obtained from all participants and/or their legal guardians. This study was performed in accordance with the Declaration of Helsinki. The study conducted in accordance with good clinical practice.

### VEMP recordings and measurements

All VEMPs were recorded using disposable silver/silver-chloride surface electrodes; abrasive gel was applied before attaching the electrodes to ensure that the impedance levels were below 2 kOhms, with a maximal side-to-side impedance difference of 1 kOhms. The ground electrode was place at Fpz.

For oVEMP recording, the active electrodes were placed symmetrically over the middle part of the lower eyelids, on top of the inferior orbital edges, and the reference electrodes were place 2 cm below these. During the measurements, subjects were asked to sit upright and to look upward at a fixed target (upward eye deviation of about 30°). The peak latencies (in milliseconds) of the N1 and P1 were measured, as were the N1-P1 peak-to-peak amplitudes (in microvolts).

For cVEMP recording, the active electrodes were placed symmetrically over the upper middle part of the sternocleidomastoid muscle bellies with the reference electrode over the sternal manubrium. The patients were asked to lift their heads up from a headrest and turn their heads away from the ear that was being stimulated. The peak latencies (in milliseconds) of the P13 and N23 were measured, as well as the P13-N23 peak-to-peak amplitude (in microvolts).

The oVEMPs and cVEMPs were elicited acoustically by means of short tone bursts with an acoustically shielded headphone (Telephonics TDH-39P, Welch Allyn, Inc.; 2 ms rise/fall and 2 ms plateau, frequency 500 Hz, 105 dB nHL). The oVEMPs and cVEMPs were recorded with a VikingQuest EMG system (VIASYS Healthcare Inc., USA). All evoked responses were amplified (5000x), band-pass filtered (30–1500 Hz), notch filtered, averaged, and recorded without artefact rejection. For each trial, 120 acoustic stimuli were averaged. We checked three trials for reproducibility.

### Statistical analysis

Data are expressed as means ± SD and frequency. Demographic and clinical variables and VEMP parameters were compared between patients with and without dizziness. The statistical significance of the demographic and clinical variables was evaluated by independent t-test and Chi-square test where appropriate. The differences in the VEMP parameters were tested by one-way analysis of covariance (ANCOVA) with age, sex, and H&Y stage as covariates. If we could not obtain VEMPs, they were recorded as "bilateral absent" or "unilateral absent", and the ratio of the three conditions (i.e., bilateral absent, unilateral absent, and present) was compared. We also performed partial correlation to assess the associations between dizziness severity and VEMP parameters in PD patients with dizziness, while controlling for age, sex, and H&Y stage. Values of p < 0.05 were regarded as significant. Statistical analysis was performed using IBM SPSS 24 Statistics (IBM Corp., Armonk, NY, USA).

## Supplementary Information

Supplementary Information 1.

Supplementary Information 2.

## Data Availability

The data that support the findings of this study are available from the corresponding author upon reasonable request.
