# Supplementary material for: Dizziness in Parkinson’s disease patients is associated with vestibular function
Source: Sci Rep. 2021 Sep 23;11:18976. doi: 10.1038/s41598-021-98540-5 (PMC8460810; doi:10.1038/s41598-021-98540-5)
Supplement: Supplementary file 1 — Supplementary Information 1. [file 41598_2021_98540_MOESM1_ESM.docx]

**Supplementary Table 1.** List of the performed neurological examination

| Evaluation component | | Neurological examination |
| --- | --- | --- |
|  | Mental status |  |
|  | All cranial nerve function | Including eye movement (saccades, pursuit, vestibular-ocular reflex), and presence of nystagmus or other ocular oscillations |
|  | Motor function | Including power, tone |
|  | Sensory function | Sense of touch, position, pain and temperature |
|  | Coordination/ataxia | Including finger-to-nose, heel-to-shin testing, rapid alternating movements, and Romberg test (to evaluate sensory ataxia) |
|  | Gait and balance | Spontaneous gait, tandem gait, and the pull test (to evaluate postural stability) |
