# Supplementary material for: Dizziness in Parkinson’s disease patients is associated with vestibular function
Source: Sci Rep. 2021 Sep 23;11:18976. doi: 10.1038/s41598-021-98540-5 (PMC8460810; doi:10.1038/s41598-021-98540-5)
Supplement: Supplementary file 2 — Supplementary Information 2. [file 41598_2021_98540_MOESM2_ESM.docx]

**Supplementary table**. Comparison of the number of patients with no dizziness and dizziness among patients with H&Y stage 2.5 or higher

|  | | Without dizziness  n=20 | With dizziness  n=15 | p-value |
| --- | --- | --- | --- | --- |
| H&Y stage | 2.5 | 14 | 13 | 0.137 |
|  | 3 | 6 | 1 |  |
|  | 4 | 0 | 1 |  |
